# The development of sensorimotor influences in the audiovisual speech domain: some critical questions

**DOI:** 10.3389/fpsyg.2014.00812

**Published:** 2014-08-06

**Authors:** Bahia Guellaï, Arlette Streri, H. Henny Yeung

**Affiliations:** ^1^Laboratoire Ethologie, Cognition, Développement, Université Paris Ouest Nanterre La Défense, NanterreFrance; ^2^CNRS, Laboratoire Psychologie de la Perception, UMR 8242, ParisFrance; ^3^Université Paris Descartes, Paris Sorbonne Cité, ParisFrance

**Keywords:** speech perception, speech production, sensorimotor systems, infants, children

## Abstract

Speech researchers have long been interested in how auditory and visual speech signals are integrated, and the recent work has revived interest in the role of speech production with respect to this process. Here, we discuss these issues from a developmental perspective. Because speech perception abilities typically outstrip speech production abilities in infancy and childhood, it is unclear how speech-like movements could influence audiovisual speech perception in development. While work on this question is still in its preliminary stages, there is nevertheless increasing evidence that *sensorimotor* processes (defined here as any motor or proprioceptive process related to orofacial movements) affect developmental audiovisual speech processing. We suggest three areas on which to focus in future research: (i) the relation between audiovisual speech perception and sensorimotor processes at birth, (ii) the pathways through which sensorimotor processes interact with audiovisual speech processing in infancy, and (iii) developmental change in sensorimotor pathways as speech production emerges in childhood.

## INTRODUCTION

A unique property of speech—compared to other auditory signals—is that it is multisensory. Speech involves not only auditory, but also visual, motor, as well as proprioceptive information, since we produce speech by moving our articulators (i.e., the jaw, tongue, lips, etc.). Accordingly, many speech researchers postulated that articulatory gestures, rather than acoustic cues, were the primary objects of speech perception (Liberman et al., 1967; Liberman and Mattingly, 1985; Fowler, 1986, 1996; Best, 1995; Galantucci et al., 2006), and in recent years, vigorous debates about these ideas have continued (Scott et al., 2009; Pulvermüller and Fadiga, 2010; Schwartz et al., 2010; Hickok, 2014). Currently, proposals suggesting that articulatory input has an important role in auditory-only speech processing (Yuen et al., 2010; Möttönen et al., 2013, 2014) have been viewed by some as highly controversial (Lotto et al., 2009; McGettigan et al., 2010; Chevillet et al., 2013).

Somewhat less controversial is the discussion of speech production in the context of *multisensory* speech processing (Ojanen et al., 2005; Skipper et al., 2007a; Okada and Hickok, 2009; Treille et al., 2014). Just as visual influences on auditory speech processing have long been reported (e.g., Sumby and Pollack, 1954; see Navarra et al., 2012 for review), recent reports have also shown similar effects from articulatory information. For example, subjects’ own silent articulations (Sams et al., 2005; Sato et al., 2013; Scott et al., 2013) influence auditory perception in similar ways as seeing visual speech (although see Mochida et al., 2013). Moreover, receiving haptic or tactile input related to another person’s articulatory movements can also influence auditory speech processing (Fowler and Dekle, 1991; Gick et al., 2008; Gick and Derrick, 2009; Ito et al., 2009; Treille et al., 2014). Neuroimaging studies converge with these behavioral findings: For example, when visual-only or audiovisual speech are presented to subjects, activation is seen in primary auditory areas of the brain, such as the superior temporal sulcus (STS), *and* in areas traditionally associated with speech production, such as Broca’s area (Calvert et al., 1997; Calvert and Campbell, 2003; Ojanen et al., 2005; Pekkola et al., 2005). TMS studies have now shown that the perception of visual and audiovisual speech is linked to primary motor cortex (Sundara et al., 2001; Sato et al., 2010), and from this accumulated evidence, there is emerging consensus that visual speech processing is closely linked to internal models of the vocal tract (Santi et al., 2003; van Wassenhove et al., 2005; Skipper et al., 2007a, b; Okada and Hickok, 2009; Dick et al., 2010; Swaminathan et al., 2013).

Here, we present a discussion of how developmental work may contribute to this broader literature. Infancy and childhood are particularly interesting because speech perception versus speech production capabilities are largely asymmetric during this period (see for reviews Oller, 1980; Stark, 1980; Werker and Yeung, 2005; Gervain and Mehler, 2010; Stoel-Gammon, 2011; Werker et al., 2012). Nevertheless, infants sometimes show neurophysiological activation that belies their apparent deficits in production. For example, areas corresponding to Broca’s area are activated in response to auditory speech even in 6 month olds (Imada et al., 2006), and while this area is also activated in a variety of adult tasks (including ones not strictly about production, see Friederici, 2012), these infant data could potentially be interpreted as reflecting rudimentary perception-production loops.

In light of infants’ limitations in the speech production domain, we use *sensorimotor* as a general term that broadly encompasses motor and proprioceptive information related to both speech-like and non-speech orofacial gestures. We focus on three issues that we see as being particularly pressing for future research: (i) the relation between audiovisual speech perception and sensorimotor processes at birth, (ii) the pathways through which sensorimotor processes interact with audiovisual speech processing in infancy, and (iii) developmental change in sensorimotor pathways as speech production emerges in infancy.

## THE RELATION BETWEEN AUDIOVISUAL SPEECH PERCEPTION AND SENSORIMOTOR PROCESSES AT BIRTH

Infants receive filtered auditory input in the womb but necessarily do not experience audiovisual speech until birth. However, as soon as it can be measured, at least some basic aspects of audiovisual perception are already present. For example, newborns map abstract sensory and magnitude information across vision and audition (Meltzoff and Borton, 1979; Streri, 1993; de Hevia et al., 2014), and it also appears that newborns are particularly sensitive to audiovisual temporal synchrony (Slater et al., 1999). The precise origin of these interactions between vision and audition remain under debate (e.g., Bahrick et al., 2004; Maurer and Mondloch, 2004; Streri, 2012), but it is clear that intersensory correspondences are powerful in that they can influence attention and learning, as shown in classic studies with precocial birds (e.g., Lickliter et al., 2002). In human newborns, temporal synchrony between audition and vision plays an important role in matching monkey faces and voices (Lewkowicz et al., 2010), and newborns’ can also match human faces and voices under some circumstances (Aldridge et al., 1999), but further research showing the mechanisms driving this matching is needed. Here we define some critical issues with regard to the role of sensorimotor processes in audiovisual processing of speech- and speech-like stimuli at birth.

It is well established that newborns imitate faces at birth, suggesting early integration of vision and proprioception (e.g., Meltzoff and Moore, 1977, 1989), although it is important to note that this has been questioned on both empirical (Anisfeld, 1996) and interpretational grounds (Jones, 2007). Still, studies using speech stimuli converge with these results. For example, newborns produce more mouth openings when listening to /a/ versus /m/ sounds, and they produce more mouth closing when listening to /m/ versus /a/ sounds (Chen et al., 2004). However, future work will need to move beyond simple correspondences between sight, sound, and movement, and ask instead how such information interacts. For example, facial imitation at birth is more robust in the presence of congruent audiovisual speech: Infants produce more mouth-opening when presented with a face saying /a/, than with the face alone, or that face dubbed with an /i/ audio track (Coulon et al., 2013). A speculative interpretation is that congruent audiovisual speech constitutes more robust input to an internal model of the vocal tract, increasing the production of relevant mouth shapes.

Another question concerns specificity: can imitation also be elicited from auditory or visual models that are *not* identifiably human, and if so, what constraints on this system exist? For example, previous work has suggested preferential processing of speech stimuli over white noise (Colombo and Bundy, 1981) and synthetic analogs of speech (Vouloumanos and Werker, 2004, 2007). However, in a striking set of studies, a preference for human over monkey vocalizations was not found at birth, but was found at 3 months of age (Shultz and Vouloumanos, 2010; Vouloumanos et al., 2010). Together, these data suggest evolutionary constraints on auditory preferences, and in turn, raise questions about the imitation studies above. Will infants produce more facial gestures in response to human versus non-human (or non-mammalian) auditory, visual, and audiovisual models? What attentional and/or evolutionary factors might drive such effects?

A final future research question must also examine the functioning of sensorimotor and perceptual systems in a more precise manner, and in more naturalistic situations. For example, recent work suggests that newborns are highly sensitive to both rigid (i.e., whole-head) and non-rigid movements (i.e., facial features) of a talking face (Guellaï et al., 2011). Do newborns privilege one type of feature over the other when imitating (see also Meltzoff and Moore, 1989)? Previous work has also shown that newborns are also more sensitive to talking faces with direct versus averted gaze (Guellaï and Streri, 2011), suggesting that foundational aspects of social communication may exist at birth. However, it remains unclear how facial imitation may change with social gaze.

## PATHWAYS THROUGH WHICH SENSORIMOTOR INFLUENCES INTERACT WITH AUDIOVISUAL SPEECH PROCESSING IN INFANCY

After the neonatal period, older infants continue to perceive audiovisual speech robustly. This has commonly been shown using a cross-modal matching procedure, where 2–4 month-olds are presented with side-by-side faces articulating the two visual vowels ([i] and [a]), accompanied by a single speech sound (either /i/ or /a/) in synchrony with both faces. Infants look longer at the matching face, showing an ability to associate vowels with the corresponding articulation (Kuhl and Meltzoff, 1982, 1984; MacKain et al., 1983; Patterson and Werker, 1999, 2002, 2003; Yeung and Werker, 2013). The effects of congruent versus incongruent audiovisual speech are also evident in a variety of other behavioral paradigms (Rosenblum et al., 1997; Burnham and Dodd, 2004; Desjardins and Werker, 2004; Pons et al., 2009; Tomalski et al., 2012; Kubicek et al., 2014; Pons and Lewkowicz, 2014), as well as in electrophysiological recordings (Kushnerenko et al., 2008; Bristow et al., 2009). A few recent papers have also begun to test audiovisual matching with fluent streams of speech (instead of just vowels or consonants; Lewkowicz and Pons, 2013; Kubicek et al., 2014), suggesting that audiovisual matching abilities in infancy can be very broad.

What about the mechanisms driving audiovisual speech perception? As mentioned above, infants at birth detect subtle differences in temporal synchrony between auditory and visual channels (Lewkowicz et al., 2010), and this is true of older infants as well (Lewkowicz, 1996, 2010). It could be that intersensory redundancy facilitates the detection of amodal properties related to vowel identity. Indeed, previous research has already shown that intersensory redundancy can enhance the detection of other kinds of amodal properties from faces (e.g., emotional affect; Flom and Bahrick, 2007), but at the cost of processing unimodal features (e.g., face identity; Bahrick et al., 2013). Together, this work suggests that synchrony detection may enhance amodal aspects of audiovisual speech (e.g., Bahrick et al., 2004).

An alternative proposal suggests that audiovisual speech information is mapped using sensorimotor information, perhaps via an internal model of the vocal tract (Kuhl and Meltzoff, 1984, 1988; Kent and Vorperian, 2007; Yeung and Werker, 2013). Several lines of evidence are suggestive of this sensorimotor mechanism: first, audiovisual matching with non-speech stimuli is often less robust than with speech (Kuhl and Meltzoff, 1984; Kuhl et al., 1991), particularly at later points in development (Lewkowicz and Ghazanfar, 2006), which suggests that audiovisual perception becomes more speech specific with age. Second, just as in newborns (Coulon et al., 2013), older infants also produce more congruent mouth shapes when hearing audiovisually congruent vowels compared to incongruent vowels (Legerstee, 1990; Kuhl and Meltzoff, 1996; Patterson and Werker, 1999). A recent report further shows that infants making /i/-like lip movements while chewing on a teething ring, or /u/-like lip movements while sucking on a pacifier, could no longer achieve match audiovisual speech matching if the heard vowel was similar the achieved lip shape (Yeung and Werker, 2013). This suggests that direct activation of the motor system can indeed affect audiovisual speech perception, and it is strongly suggestive of sensorimotor influences.

Together, this work raises two critical areas of future research. First, these dueling approaches must be reconciled: Are auditory and visual speech are bound together by temporal synchrony cues, or is there some internal model of the vocal tract that accomplishes this mapping? A third alternative is that two separate modes of audiovisual processing will be identified. For example, recent work has suggested that synchrony detection in 5 month-old infants uses a fast and automatic pathway which could be similar to the kind of adult audiovisual pathways that activate the STS and its associated networks (Hyde et al., 2011). More work is needed to see whether a slower, higher level pathway can also be distinguished, and if this pathway also taps sensorimotor information.

A second question concerns the definition of orofacial movements in infancy. Some work suggests that early vocalizations can already be considered speech-like: Cooing and babbling are influenced by the phonological properties of the native language (De Boysson-Bardies et al., 1989; Ruzza et al., 2006; Whalen et al., 2007), and are argued to be continuous with the first productions of words (de Boysson-Bardies and Vihman, 1991; Vihman, 1991; McCune and Vihman, 2001). Infant vocalizations also change in response to socially contingent responses from mothers, whether manipulated in the laboratory (Goldstein and Schwade, 2008), or measured during free play (Gros-Louis et al., 2014). Other work has even suggested that babbling capacities act as an attentional filter on auditory speech perception, modulating preferences to listen to words that either share or do not share commonalities between what is produced in babbling and in one’s early words (DePaolis et al., 2011, 2013; Majorano et al., 2014). At the same time, other research argues instead that universal constraints on the motor system (not specific to speech) play an equally important role in structuring how babbling is produced (MacNeilage and Davis, 1993; Lee et al., 2010). Moreover, coordinative movements differ when infants speak, babble, suck, or chew, suggesting that the physical mechanisms underlying babbling are not continuous with later speech motor control (Steeve, 2010).

In conjunction with the results from Yeung and Werker (2013), which demonstrate an effect of non-speech movements, the above debate shows how difficult it is to define what counts as an articulatory (i.e., speech-like) gesture, which in turn makes it hard to speculate about how an internal model of the vocal tract might be structured in early development (although see Ménard et al., 2007; Howard and Messum, 2011). Future research postulating a sensorimotor pathway in infancy will need to bear this literature in mind. One intriguing possibility is that distinctions between “speech-like” or “non-speech-like” may not be important at all (at least in early development): For example, infants have more difficulties matching auditory whistles to visual faces that are whistling than auditory trills to visual faces that are trilling. One speculative reason for this asymmetry could be that infants *produce* bilabial trills, but do not yet produce whistles (Mugitani et al., 2008).

## DEVELOPMENTAL CHANGE AS SPEECH PRODUCTION BECOMES MORE VARIED AND SOPHISTICATED

Of course, the development of perceptual and motor systems continues well beyond infancy. For example, previous reports show that children (up to the age of 10) increasingly weight visual speech information more heavily in cases of sensory conflict or ambiguity (McGurk and MacDonald, 1976; Massaro, 1984; Massaro et al., 1986; Wightman et al., 2006; van Linden and Vroomen, 2008; Barutchu et al., 2010; Ross et al., 2011). It seems likely that multiple mechanisms drive this developmental change: For example, Sekiyama and Burnham (2008) find cross-cultural differences, which are likely unrelated to differences in motor ability. Nevertheless, there is also some correlational evidence supporting a sensorimotor pathway: children who have greater trouble articulating consonants show less sensitivity to visual speech information (Desjardins et al., 1997), as is also the case for children with broader language deficits (Bergeson et al., 2005; Dodd et al., 2008).

Other studies provide further evidence for multiple pathways emerging in childhood that are reminiscent of adult models (e.g., Skipper et al., 2007b; Okada and Hickok, 2009; Hickok et al., 2011). For example, while visual speech is more heavily weighted throughout childhood, non-speech audiovisual processing is relatively stable (Tremblay et al., 2007; although see Hillock et al., 2011). Such divergent trajectories suggest that two kinds of audiovisual binding mechanisms may be dissociated. Neurophysiological evidence for that dissociation comes from a study revealing two separable electrophysiological measures: amplitude versus latency of the commonly evoked N1/P2 complex to audiovisual speech (Knowland et al., 2014). Critically, only amplitude changes in development, while latency remains stable. Additional evidence comes from functional imaging studies, which suggests two networks related to audiovisual binding of speech stimuli: One network is centered around primary auditory areas, while a second network involves inferior frontal areas (Dick et al., 2010; Nath et al., 2011). Developmental change in audiovisual speech processing correlates with changes in connectivity between these networks (Dick et al., 2010).

Together these findings are highly suggestive of at least two distinct pathways in the brain that support audiovisual speech processing. A preliminary conjecture is that multiple pathways might be distinguished based on their developmental characteristics (stable, or increasing), their selectivity (to speech, or to may kinds of signals), and their mechanisms (depending on intersensory redundancy, or depending on an internal articulatory model). Critical lines of future research will need to investigate these hypotheses.

## CONCLUSION

Speech perception is one of the most deeply explored aspects of language development. However, as this review highlights, several aspects of this phenomenon remain mysterious: in particular, the relation between speech perception and production. Here, we examine possible sensorimotor influences in multisensory speech processing, highlighting three areas for future research that will bridge between debates in the adult literature and emerging work in development.

First, we suggest that future research must examine the link between imitation and audiovisual speech perception at birth, and explore interactions among vision, audition, and the motor system. Second, we highlight two potential pathways involved in audiovisual speech perception in older infants, one of which may depend on sensorimotor information. Third, we illustrate the need to elucidate the behavioral and at the neural characteristics of these pathways in children, as speech production becomes more sophisticated.

## Conflict of Interest Statement

The Guest Associate Editor Maya Gratier declares that, despite being affiliated to the same institution as author Bahia Guellaï, the review process was handled objectively and no conflict of interest exists. The authors declare that the research was conducted in the absence of any commercial or financial relationships that could be construed as a potential conflict of interest.
